# Solvothermal Synthesis, Structural Characterization and Optical Properties of Pr-Doped CeO_2_ and Their Degradation for Acid Orange 7

**DOI:** 10.3390/ma15196953

**Published:** 2022-10-07

**Authors:** Yaohui Xu, Pingkeng Wu, Mingjin Wu, Yuehe Gu, Hongguang Yu, Zhao Ding

**Affiliations:** 1 Laboratory for Functional Materials, School of New Energy Materials and Chemistry, Leshan Normal University, Leshan 614004, China; 2 Department of Chemical Engineering, Illinois Institute of Technology, Chicago, IL 60616, USA; 3 National Engineering Research Center for Magnesium Alloys, College of Materials Science and Engineering, Chongqing University, Chongqing 400044, China

**Keywords:** CeO_2_, Pr-doping, photocatalysis, pollutant degradation, mesoporous structure

## Abstract

Pr-doped CeO_2_ with different doping levels was prepared from Ce(NO_3_)_3_∙6H_2_O and Pr(NO_3_)_3_∙6H_2_O by solvothermal method without any additional reagents, in which the mixed solution of ethylene glycol and distilled water was employed as a solvent. The influences of Pr-doping on phase composition, crystal structure and morphology were investigated, as well as Pr valence and oxygen vacancy defects. The Pr cations entered into the CeO_2_ crystal lattice with normal trivalence and formed a Pr-CeO_2_ solid solution based on the fluorite structure. The larger trivalent Pr was substituted for tetravalent Ce in the CeO_2_ crystal and compensated by oxygen vacancy defects, which caused the local lattice expansion of the crystal lattice. Moreover, the Pr-doped CeO_2_ solid solutions exhibited visible color variation from bright cream via brick red to dark brown with the increasing of Pr contents. The degradation of AO7 dye was also investigated using a domestic medical ultraviolet lamp; the removal efficiency of AO7 by 1% and 2% Pr-doped CeO_2_ approached 100%, much higher than 66.2% for undoped CeO_2_.

## 1. Introduction

Cerium (Ce) is the most abundant and cheapest rare earth element in nature [1], and its stable oxide (ceria, CeO_2_) has special electronic configurations and abundant energy level structures, which has been widely used in various traditional and high-tech technologies, such as an oxidant [2], UV blocking agent [3], catalysts for organic reactions [4], catalyst carrier [5], glass polishing powder [6], glass decolorizing and coloring agent [7] and so on.

Energy and the environment are two major problems facing mankind today [8,9,10,11,12]. At present, the water pollution caused by azo dyes has caused widespread concern because of their carcinogenic risks and difficult biodegradation [13,14]. So far, numerous methods have been developed to remove azo dyes from contaminated water, such as adsorption technique [15], membrane separation technique [16], biofilm process [17], coagulation-flocculation precipitation [18], activated sludge method [19] and oxidation reduction method [20]. Among these available chemical and physical methods, the photocatalysis using semiconductor materials as catalysts for solar energy conversion and environmental protection has received extensive attention in recent years [21,22,23]. The greatest advantages of the photocatalytic process consist of not only the general mild reaction conditions, but also the possibility to abate refractory, toxic and difficult or non-biodegradable organic molecules [24,25]. Titanium dioxide (TiO_2_) is the most studied and widely used photocatalyst in the removal of organic dyes because of its non-toxicity, low-cost and excellent photocatalytic properties [26,27]. CeO_2_, an N-type semiconductor, is one of the more active, versatile and inexpensive rare earth oxides [28], which should be an alternative candidate photocatalyst due to the readily available intrinsic oxygen vacancies in CeO_2_ crystal. However, the use of CeO_2_ as the main active component or as a single catalyst for the photocatalytic degradation of azo dyes is really just beginning. For example, Foletto [29] et al. synthesized the CeO_2_-SnO_2_ nanocomposites with different CeO_2_ contents by a coprecipitation process. The CeO_2_-SnO_2_ composite with 7 wt.% CeO_2_ showed the highest photocatalytic activity for the degradation of Direct Black 38 (DB38) dye under sunlight, and its catalytic activity was similar to that of the commercially available TiO_2_ (Degussa P25). Mousavi [30] et al. also investigated the contribution of a nanofiber-based support and dendrimer coating on the catalytic activity of CeO_2_ nanoparticles towards phenol and azorubine dye under both UV and visible light illumination. The results indicated that these CeO_2_ nanoparticles possessed the capability to become a visible light photocatalyst with the support of the electrospun nanofiber mats. Moreover, Mishra [31] et al. synthesized the well-dispersed CeO_2_ nanoparticles by a microwave-assisted hybrid hydrothermal method and used them for simultaneous adsorption/photocatalytic decolourisation of Alizarin Red S (ARS) and Eriochrome Black-T (EBT) dyes. The monolayer adsorption capacities of ARS and EBT dyes on CeO_2_ nanoparticles were 44.1 and 57.8 mg/g without light irradiation, respectively. In addition, the overall decolourisation (adsorption/photo-degradation) of both ARS and EBT dyes (100 mg/L) increased substantially, and the optimum amounts of CeO_2_ required for ARS and EBT were 0.80 and 0.60 g/L, respectively. Despite this progress in the successful synthesis of CeO_2_ or their composites, it is still challenging to further improve their adsorption/photo-degradation capacity since the high band-gap energy of CeO_2_ restrains the utilization of the solar spectrum. For that, doping of CeO_2_ with other metal ions was presented, which performed by substituting lower valence cations into CeO_2_ lattice and introducing an oxygen vacancy defect to maintain overall charge neutrality.

Keeping this in mind, we chose a trivalent praseodymium cation (Pr^3+^; 0.1126 nm) with similar ionic radii to Ce^4+^ (0.097 nm) as the cation dopant based on the similarity–intermiscibility theory, and the Pr-doped CeO_2_ solid solutions by solvothermal method for simultaneous adsorption/photocatalytic degradation of Acid Orange 7 dye (AO7) were proposed. To date, many methods had been developed to synthesize Pr-doped CeO_2_, such as the Sol-Gel method [32], self-propagating method [33] and conventional solid-state reaction method [34]. Considering the advantages of simple operation and low energy consumption of the solvothermal method, a series of Pr-doped CeO_2_ with different Pr contents was prepared solvothermally from Ce(NO_3_)_3_∙6H_2_O as a cerium source, Pr(NO_3_)_3_∙6H_2_O as a dopant and the mixed solution of ethylene glycol and distilled water as a solvent in this work. The characterizations of the phase composition, lattice parameters, grain sizes, Pr valence, oxygen vacancy defects, specific surface area, morphology, light absorption ability and subjective color change of the as-prepared Pr-doped CeO_2_ were investigated and discussed. Subsequently, the decolourisation (adsorption/photo-degradation) of AO7 dye was also investigated using a domestic medical ultraviolet lamp.

## 2. Experimental

### 2.1. Starting Materials

Ce(NO_3_)_3_∙6H_2_O (99.95%) and Pr(NO_3_)_3_∙6H_2_O (99.99%) were supplied by Aladdin Co. Ltd., Ontario, CA, USA; ethylene glycol (99.5%) and ethanol (99.7%) were supplied by Chengdu Kelong Chemical Co. Ltd., Chengdu, China. And Acid Orange 7 (AO7, 97.0%) was supplied by Tokyo Chemical Industry Co. Ltd., Tokyo, Japan. All chemicals were used as received without further purification.

### 2.2. Synthesis of Undoped and Pr-Doped CeO_2_

A series of Pr-doped CeO_2_ was prepared with different molar concentrations of Pr cation through a solvothermal method combined with subsequent calcination in air, in which Ce(NO_3_)_3_∙6H_2_O served as cerium source, whereas Pr(NO_3_)_3_∙6H_2_O served as dopant. Typically, appropriate amounts of Ce(NO_3_)_3_∙6H_2_O and Pr(NO_3_)_3_∙6H_2_O with a total of 4.0 mmol were dissolved in a mixed solution of 25 mL ethylene glycol and 5 mL distilled water, and then the above-mentioned mixed solution was decanted into a 50 mL Teflon-lined stainless steel autoclave and sealed. After maintaining the solution at 200 °C for 24 h, the precursor powders were collected by centrifugation and washed with distilled water and ethanol, and then dried in air at 60 °C for 24 h. Finally, a series of Pr-doped CeO_2_ powders was obtained by subsequent calcination in air at 500 °C for 2 h. The as-prepared Pr-doped CeO_2_ powders with different molar concentrations of Pr were labeled as 1% Pr-doped CeO_2_, 2% Pr-doped CeO_2_, 3% Pr-doped CeO_2_, 4% Pr-doped CeO_2_, 5% Pr-doped CeO_2_ and 6% Pr-doped CeO_2_. Similarly, the undoped CeO_2_ powders were also prepared following the same procedure as control; however, in the absence of Pr(NO_3_)_3_∙6H_2_O, it was labeled as Undoped CeO_2_.

The practical contents of the Pr element in CeO_2_ were measured by ICPMS and are shown in Table 1; the practical Pr contents in CeO_2_ were close to the corresponding nominal doping concentrations.

### 2.3. Characterization

The crystallographic phases of cerium precursors and CeO_2_ samples were characterized by X-Ray Diffraction (XRD, DX-2700) analysis. The surface compositions and binding energies of CeO_2_ samples were determined by X-ray Photoelectron Spectroscopy (XPS, ESCALAB 250Xi). The practical doping levels of Pr elements in CeO_2_ samples were determined using Inductive Coupled Plasma Mass Spectrometry (ICPMS, Agilent-7800, Agilent Technologies, Carpinteria, CA, USA). The morphologies of CeO_2_ samples were evaluated by field-emission Scanning Electron Microscopy (SEM, JEOL-7500F, JEOL Ltd., Tokyo, Japan). N_2_ adsorption–desorption isotherms were measured using a QuadraSorb SI surface area analyzer, and the BET specific surface areas were determined using the Brunauer–Emmett–Teller method. Raman spectra were obtained using the LabRAM HR800 (Jobin Yvon Co., Paris, France) with a 325 nm He-Cd laser. The reflectance spectra of CeO_2_ samples, UV-VIS absorption spectra and absorbances of AO7 solutions were measured using an Ultraviolet-Visible spectrophotometer (UV-VIS, U-3900, Hitachi Ltd., Tokyo, Japan).

### 2.4. Photoreactor and Light Source

All batch experiments were performed in a 150 mL ceramic plate (Φ18 cm) at room temperature without irradiation or under irradiation by a 300 W ultraviolet lamp (300 W; 254 nm). Such ultraviolet radiation was generated by a medical ultraviolet disinfection vehicle (220 V, DANCHENG, China). During the reaction, the air conditioning was turned on to ensure the room temperature was 25 °C. The adsorption in dark and photocatalysis experiments were performed without any stirring, additional oxidant or any other substance. In addition, the distance between the light source and the liquid level was 15 cm.

### 2.5. Degradation of AO7 Dye

The adsorptive/photocatalytic degradations of AO7 dye on Pr-doped CeO_2_ were evaluated both in the dark and under UV light illumination using a medical ultraviolet lamp. Typically, 0.1 g CeO_2_ was dispersed into 100 mL AO7 solution (20 mg/L). Before illumination, it was left to stand for 1 h in the dark, and then the mixture was exposed to the ultraviolet radiation. Then, about 5 mL suspensions were withdrawn at regular intervals of 0.5 h and separated by centrifugation, and then the absorbance of supernatant was measured immediately at the absorption wavelength of 485 nm using a U-3900 spectrophotometer. During the test of absorbance, the adsorption or photocatalytic experiment was suspended and stopped; specifically, the heterogeneous photocatalysis reaction system was covered with a shade cloth. After the measurement of absorbance, the taken suspension including CeO_2_ and AO7 solution were put back into the original photocatalytic system, and the photocatalytic experiment was restarted. Finally, the removal rate (*η*, %) was estimated using Equation (1).

(1) $$\eta_{}(\%)=\frac{A_{0}-A_{t}}{A_{0}}\times 100$$

where *A*_0_ is the absorbance of the initial AO7 solution (20 mg/L) and *A*_t_ is the absorbance of the AO7 solution at a given time *t*.

## 3. Results and Discussion

**Figure 1:**
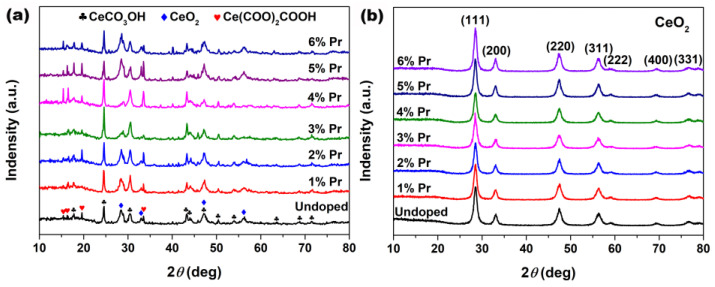
XRD patterns of the samples synthesized solvothermally at 200 °C for 24 h with different Pr concentrations (**a**) before and (**b**) after calcination in air at 500 °C for 2 h.

XRD was employed to research the effect of the introduction of Pr elements on the phase composition and crystallographic structure of the samples. Figure 1a shows XRD patterns of the cerium precursors synthesized with different Pr concentrations by solvothermal treatment at 200 °C for 24 h. As observed in Figure 1a, all precursors displayed a mixture of CeCO_3_OH (JCPDS no. 52-0352, Hexagonal), Ce(COO)_2_COOH (JCPDS no. 51-0548, Orthorhombic) and CeO_2_ (JCPDS no. 34-0394, Cubic) characteristic peaks. After the following calcination in air at 500 °C for 2 h, all samples in Figure 1b displayed several well-resolved XRD peaks that were indexed to the (111), (200), (220), (311), (222), (400) and (331) planes of fluorite CeO_2_ (JCPDS no. 34-0394, Cubic); the peaks related to CeCO_3_OH and Ce(COO)_2_COOH were no longer present and no additional phases from impurities, such as Pr_2_O_3_, were detected. Moreover, the grain sizes of these particles were estimated using Scherrer’s formula based on XRD patterns and are summarized in Table 1. As observed, Pr-doping had a certain inhibitory effect on the growth of CeO_2_ grains, especially 3% Pr-doped CeO_2_; its grain size was only 10.4 nm, much smaller than 16.3 nm for Undoped CeO_2_.

The calculated values of lattice parameters were measured based on Bragg’s equation and are summarized in Table 1. As observed, the calculated lattice parameters for Pr-doped CeO_2_ synthesized with different Pr contents were greater than that of Undoped CeO_2_, which could be attributed to the substitution of Ce^4+^ (0.097 nm) ions with the larger Pr^3+^ (0.1126 nm [35]) ions, and the local lattice expansion of CeO_2_ crystal occurred as a result. In addition, the lattice parameter of doping CeO_2_ reached a maximum with a Pr content of 3%, decreasing with higher Pr contents. So, the concentration (3%) could represent the solid solubility limit of Pr cation in CeO_2_ lattice. These findings indicated that the as-prepared CeO_2_ samples could maintain the cubic fluorite structure with Pr-doping, and the large Pr cations partially substituted the Ce ions to form a solid solution with a solid solubility limit of 3%.

**Figure 2:**
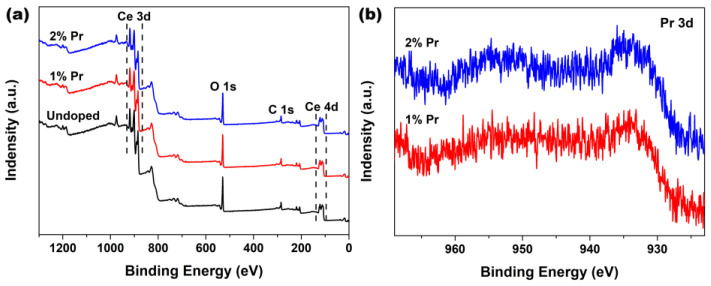
(**a**) XPS spectra of Undoped, 1% and 2% Pr-doped CeO_2_ and (**b**) the corresponding XPS regions of Pr3d of 1% and 2% Pr-doped CeO_2_.

In order to probe the surface chemical compositions and their oxidation states that might have occurred in the Pr-doping CeO_2_ systems, XPS analysis was employed to study the Undoped and Pr-doped CeO_2_ samples. Figure 2a shows the wide-scan spectra of Undoped, 1% and 2% Pr-doped CeO_2_. As observed, all of these wide-scan spectra were dominated by the signals of Ce3d, Ce4d, O1s and C1s, and their profiles were similar to that obtained in the previous studies for CeO_2_ [36]. Moreover, the corresponding Pr3d XPS regions of 1% and 2% Pr-doped CeO_2_ were recorded and are shown in Figure 2b. The characteristic peaks of Pr3d XPS regions implied that Pr were in +3 states, indicating that the Pr element had been successfully incorporated into the CeO_2_ lattice with normal trivalence states [37,38,39,40].

**Figure 3:**
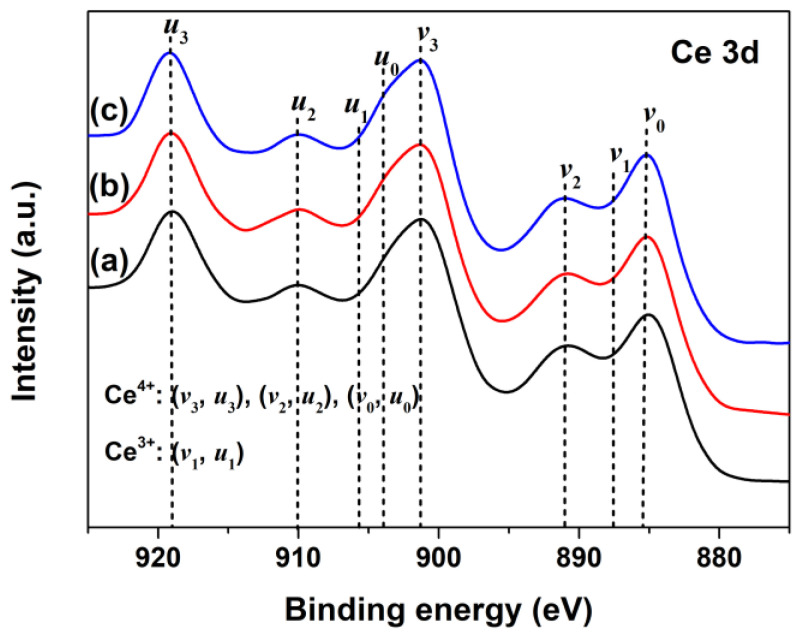
Ce3d XPS spectra of (**a**) Undoped, (**b**) 1% Pr- and (**c**) 2% Pr-doped CeO_2_.

To investigate the changes of oxidation states of Ce in CeO_2_ with Pr doping, the Ce3d XPS regions of Undoped, 1% and 2% Pr-doped CeO_2_ were recorded and are shown in Figure 3a–c, respectively. According to previous research [41], the Ce3d XPS peak of CeO_2_ could be divided into eight separate peaks, referring to the 3d_5/2_ and 3d_3/2_ spin–orbit component of cerium cations; the bonds labeled as *v*_1_ and *u*_1_ belonged to the unique photoelectron features from Ce^3+^ states, whereas the bands labeled as *v*_3_, *v*_2_, *v*_0_ (and those for *u*) were due to Ce^4+^ states. As observed in Figure 3a, the Ce3d XPS region of Undoped CeO_2_ contained five peaks only, and no peaks associated with Ce^3+^ species (*v*_1_ and *u*_1_) were found, which was consistent with the previous report of Ce^4+^ cations, indicating the main valence of cerium in Undoped CeO_2_ was +4 [42]. Compared to the Ce3d XPS spectrum of Undoped CeO_2_ in Figure 3a, no significant changes in peak shape and binding energy were observed for 1% and 2% Pr-doped CeO_2,_ as Figure 3b,c show. These findings indicated that the Pr-doping had little effect on the production of Ce^3+^ species in CeO_2_; in other words, Ce in the CeO_2_ surface was still dominated by Ce^4+^ after Pr-doping.

**Figure 4:**
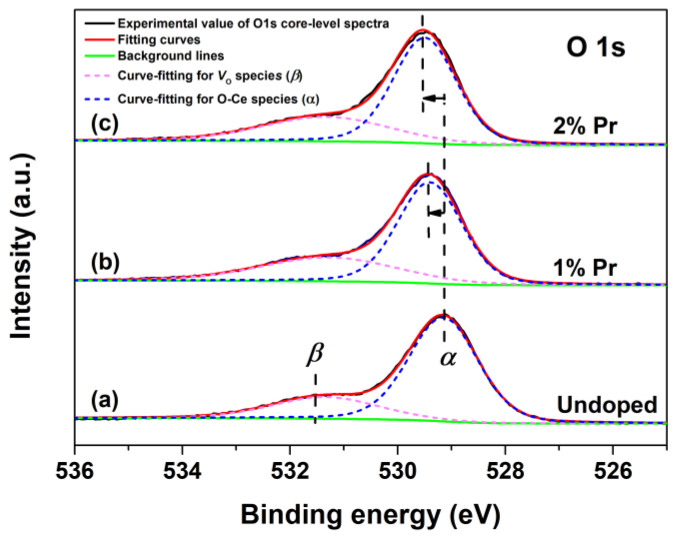
O1s core-level XPS spectra of (**a**) Undoped, (**b**) 1% and (**c**) 2% Pr-doped CeO_2_.

To investigate the changes of chemical states of oxygen in CeO_2_ with Pr doping, the O1s XPS regions and respective fitting curves of Undoped, 1% and 2% Pr-doped CeO_2_ were recorded, and the results are shown in Figure 4. The O1s XPS spectra of Undoped CeO_2_ could be curve-fitted into two peaks, indicative of the presence of two kinds of oxygen species on the CeO_2_ surface. The peak at about 529.1 eV (labeled as *α*) could be assigned to the lattice oxygen of O-Ce species, whereas that of about 531.5 eV (labeled as *β*) could be assigned to the chemisorbed oxygen species or/and weekly bonded oxygen species related to the oxygen vacancies (labeled as *V*_O_). The *β* peak that appeared in Undoped CeO_2_ indicated that pure CeO_2_ itself possessed a certain number of *V*_O_ defects. Considering the traces of the Pr element in doping CeO_2_, the O1s XPS spectra of 1% and 2% Pr-doped CeO_2_ were also curve-fitted into two peaks. Compared to the binding energy of the O1s spectrum in Undoped CeO_2_, there was a blue shift of 0.3 eV for 1% Pr-doped CeO_2_ and 0.4 eV for 2% Pr-doped CeO_2_, respectively. The blue shift of O1s peaks suggested the reduction of the valence state of lattice oxidation from O-Ce species; in other words, Pr-doping benefited the *V*_O_ species in creation. The rising numbers of *V*_O_ species in CeO_2_ could be attributed to the incorporation of Pr^3+^ into the CeO_2_ lattice to form solid solutions. The substitution reaction of Ce^4+^ cation by Pr^3+^ cation could be written in Kroger and Vink notations, as expressed by Equation (2):

(2) $${\mathrm{Pr}}_{2}O_{3}\;\overset{{2\mathrm{CeO}}_{2}}{⇄}{\;2\mathrm{Pr}}_{\mathrm{Ce}}^{’}+3O_{O}^{\times }+V_{O}^{••}$$

where ${\mathrm{Pr}}_{\mathrm{Ce}}^{’}$ represents a Pr^3+^ cation occupying the site of a Ce^4+^ cation, $O_{O}^{\times }$ is a lattice oxygen atom, and $V_{O}^{••}$ represents an oxygen vacancy with two positive charges. In addition, the oxygen vacancies ratio (labeled as [*V*_O_]_XPS_) could be quantified using Equation (3), and the calculated values are shown in Table 1.

(3) $${\left[V_{O}\right]}_{\mathrm{XPS}}(\%)=\frac{A_{\beta }}{A_{\alpha }+A_{\beta }}\times 100$$

where [*V*_O_]_XPS_ (%) represents the relative oxygen vacancy concentration calculated by O1s XPS spectra and *A*_α_ and *A*_β_ are the integrated area of Peak *α* and Peak *β* from the O1s core-level XPS spectra in Figure 4. As shown in Table 1, the estimated values [*V*_O_]_XPS_ of 1% and 2% Pr-doped CeO_2_ were 33.30% and 31.67%, respectively, higher than that of Undoped CeO_2_ (24.36%), which further indicated that Pr-doping promoted the creation of *V*_O_ species in CeO_2_. Further analysis of the relative *V*_O_ concentration was conducted by Raman spectra analysis, as will be discussed later.

**Figure 5:**
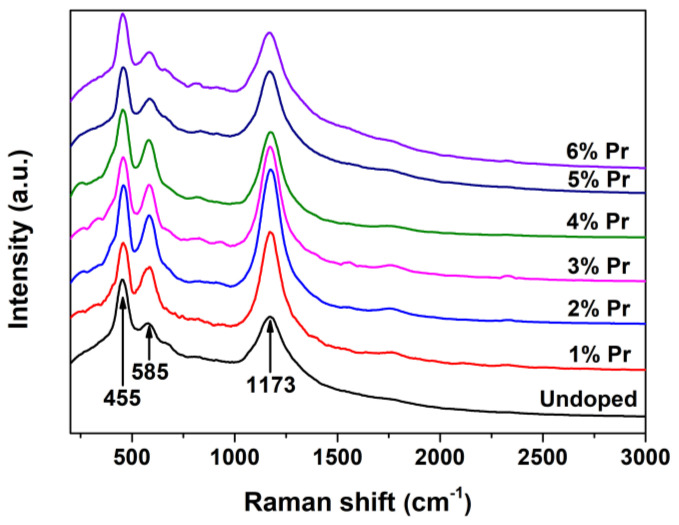
Raman spectra of Undoped CeO_2_ and Pr-doped CeO_2_ with different Pr contents.

Laser Raman is rather powerful in identifying the nature of surface *V*_O_ defects because of its sensitivity to crystalline symmetry [43]. The Raman spectra for Undoped and Pr-doped CeO_2_ are shown in Figure 5. As observed, the undoped CeO_2_ showed an obvious band at 455 cm^-1^, which can be assigned to the F_2g_ vibration mode of the O atoms around Ce^4+^ cations, and the band at 1173 cm^-1^ can be attributed to the second-order transverse acoustic mode of CeO_2_ fluorite structure, whereas the weak band at 585 cm^-1^ has been proven to be associated to the *V*_O_ defects and has been widely observed in substoichiometric CeO_2-x_ [44]. The presence of the weak band at 585 cm^-1^ suggested that there existed a certain amount of *V*_O_ defects in pure CeO_2_. Moreover, the Pr-doped CeO_2_ exhibited a stronger 585 cm^-1^ peak than the undoped one; the intensity of the band at 585 cm^-1^ initially increased and then decreased with the increasing Pr-doping amount. As an alternative approach to estimate the concentration of *V*_O_ defects in CeO_2_, the intensity ratio of the bands at 585 and 455 cm^-1^ (*I*_585_/*I*_455_, labeled as [*V*_O_]_Raman_) was calculated, and the results are shown in Table 1. The relative [*V*_O_]_Raman_, which is the value of *I*_585_/*I*_455_, reached a maximum in 1% Pr-doped CeO_2_, then decreased.

**Figure 6:**
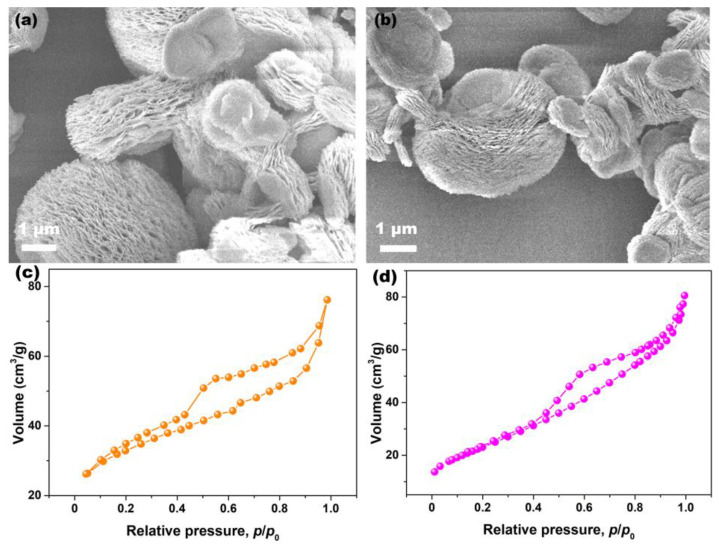
SEM images of (**a**) Undoped and (**b**) 6% Pr-doped CeO_2_, N_2_ adsorption–desorption isotherms of (**c**) Undoped and (**d**) 6% Pr-doped CeO_2_.

To clarify the effects of Pr-doping on the morphology of CeO_2_, SEM analysis was conducted. Figure 6a,b show the SEM images of Undoped and 6% Pr-doped CeO_2_ particles. As observed, both the morphologies of Undoped and Pr-doped CeO_2_ particles were the multilayered structure consisting of nanoflakes that intercalated, forming an open, porous structure. To further clarify the porous nature of CeO_2_ samples, N_2_ adsorption–desorption experiments were conducted, and their BET specific surface areas were determined using the Brunauer–Emmett–Teller method. Figure 6c,d show the N_2_ adsorption–desorption isotherms of Undoped and 6% Pr-doped CeO_2_, respectively. The hysteresis loops in the relative pressure (*p*/*p*_0_) range of 0.4–1.0 were observed, consistent with type IV hysteresis loops, confirming their mesoporous structure [45]. Moreover, the BET specific surface area of Undoped CeO_2_ was 96.0 m^2^/g, slightly higher than that of 6% Pr-doped CeO_2_ (85.8 m^2^/g). Compared to Undoped CeO_2_, the morphology of 6% Pr-doped CeO_2_ still maintained the original multilayered structure, implying that the incorporation of Pr cations into the CeO_2_ lattice had little impact on their morphology; however, Pr-doping could affect their specific surface areas.

**Figure 7:**
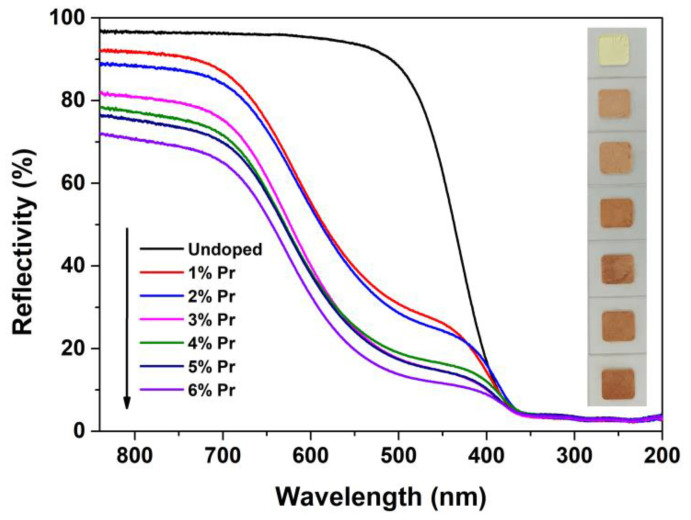
Reflectance spectra and photographs of Undoped CeO_2_ and Pr-doped CeO_2_ with different Pr contents.

UV-VIS spectroscopic measurements were carried out to investigate the effect of Pr-doping and their concentrations on the optical properties of CeO_2_. Figure 7 shows the reflectance spectra of Undoped and Pr-doped CeO_2_ with different Pr contents. For the reflectance spectrum of Undoped CeO_2_, no visible absorption was detected in the wavelength region of 840–520 nm, but strong absorption in the wavelength region of 350–200 nm was observed. This ultraviolet absorption was due to the charge–transfer transition from 2*p* orbitals of O^2-^ of the valence band (VB) to 4*f* orbitals of Ce^4+^ of the conduction band (CB) [46,47]. Compared with the reflectance spectrum of Undoped CeO_2_, 1% and 2% Pr-doped CeO_2_ showed greater absorption in the wavelength region of 840–400 nm, and higher concentration of Pr-doping (3 to 6%) could result in stronger absorption. This was due to the reduction of band gaps between VB and CB caused by the introduction of Pr^3+^ cation into CeO_2_ crystal. Moreover, the colors of Undoped and Pr-doped CeO_2_ with different Pr contents are also shown in Figure 7. As observed, the color of Undoped CeO_2_ appeared as bright cream because of its major absorption in the wavelength region of 350–200 nm, and Pr-doped CeO_2_ exhibited a visible color variation from bright cream via brick red to dark brown with the increasing of Pr contents, which could be due to the doping of Pr^3+^ cations into the CeO_2_ lattice that reduce the optical band gap value between VB and CB.

**Figure 8:**
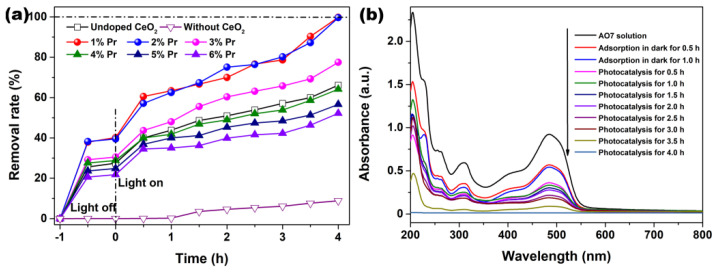
(**a**) AO7 adsorption in dark and photocatalytic degradation upon illumination using an ultraviolet lamp (300 W; *λ* = 254 nm) in the presence of Undoped CeO_2_ and Pr-doped CeO_2_ with different Pr concentrations, (**b**) UV-VIS absorption spectra of AO7 removal by 1% Pr-doped CeO_2_. ([AO7] = 20 mg/L; [CeO_2_] = 1.0 g/L; *V* = 100 mL; Room temperature; Without pH preadjustment).

The adsorption in dark and photocatalytic degradation of AO7 solutions by Pr-doped CeO_2_ were measured and compared with that of Undoped CeO_2_. Figure 8a shows the removal rates of AO7 in the presence of Undoped and Pr-doped CeO_2_. In order to make sure of the important role of CeO_2_ and Pr-doped CeO_2_, a blank experiment (self-photosensitized process) was also performed under identical conditions. As observed in Figure 8a, the blank test confirmed that AO7 dye only slightly was degraded under ultraviolet light in the absence of CeO_2_ and Pr-doped CeO_2_, indicating that the photolysis could be ignored. Like the adsorption of AO7 in the dark, all CeO_2_ samples nearly reached an adsorption–desorption equilibrium within 30 min of reaction; however, only 1% and 2% Pr-doped CeO_2_ (37.9% and 38.2%) possessed the remarkable adsorption capability of AO7 compared to that of Undoped CeO_2_ (25.6%). After 4 h of reaction, the removal rates of AO7 by 1% and 2% Pr-doped CeO_2_ approached 100%, much higher than 66.2% for Undoped CeO_2_. From the analysis results of Raman spectra in Figure 5, Pr-doping with a 1–6% level could induce more *V*_O_ defects than Undoped CeO_2_. The number of *V*_O_ basically stayed the same with the doping concentration more than 3%, but their colors got darker and darker, which changed the absorption and utilization of light, supported by UV-VIS spectra in Figure 7. In addition, Pr-doping had different effects on the BET specific surface area and grain size of CeO_2_. Therefore, we could draw a conclusion that the factors affecting the photocatalytic efficiency of CeO_2_ for AO7 were not single, and it was believed that the changes in the grain size [*V*_O_] and the optical properties caused by doping effects with Pr had affected the adsorption capability and photocatalytic activity of AO7.

The continuous UV-VIS spectra of the centrifuged solution after adsorption and catalytic reactions at the different intervals were used to record and contrast with that of the initial AO7 solution, which further clarified the removal performance and mechanism of AO7. As observed in Figure 8b, the absorption spectrum of the original AO7 solution was characterized by one main band in the visible region with its maximum absorption at 485 nm, corresponding to the azo bond (-N=N-) of the AO7 molecule, which was responsible for the orange-red color of the aromatic rings pertaining to the azo groups. Other bands in the ultraviolet region were attributed to the benzene ring and naphthalene ring structure of the AO7 molecule [48]. Figure 8b shows the UV-VIS absorption spectra of AO7 removal by 1% Pr-doped CeO_2_. As observed, the removal rate in Figure 8a increased with respect to the treatment time, and, correspondingly, the amplitude of all peaks in Figure 8b decreased with respect to time. The continuous decrease of the absorbance peak at 485 nm and other bands in the ultraviolet region suggested that the azo bonds and the naphthyl rings of the AO7 molecule were destroyed. After photocatalysis for 4.0 h, the major absorption peaks of AO7 dye in UV-VIS region had basically disappeared. UV light illumination of AO7 aqueous solution in the presence of the as-synthesized Pr-doped CeO_2_ caused the absorption bands of AO7 dye in the visible region to decrease with time and finally to disappear, suggesting the destruction of its chromophoric structure in the vicinity of the azo linkage. Moreover, no other additional absorption bands were detected from Figure 8b, such as toxic organic by-products during the photocatalytic degradation of AO7, suggesting the superiority of photocatalytic degradation of organic dyes.

**Figure 9:**
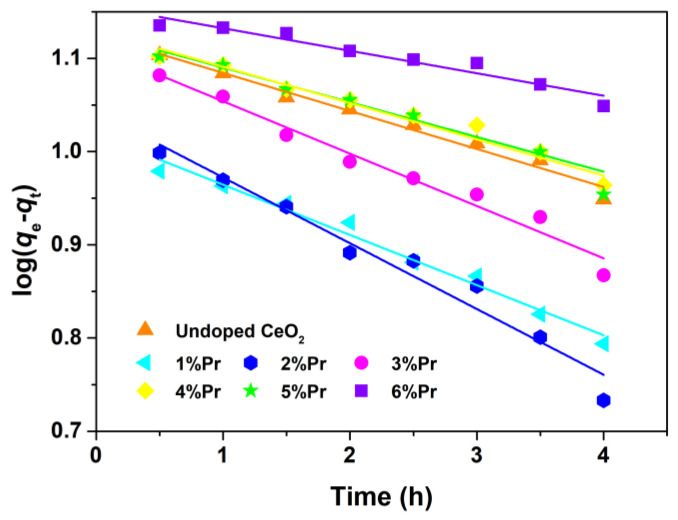
Kinetic fitting for the degradation of AO7 dye with Undoped CeO_2_ and Pr-doped CeO_2_ under ultraviolet lamp (300 W; *λ* = 254 nm).

The photocatalytic degradation reaction could be assumed to follow a pseudo-first-order kinetic expression, as expressed by Equations (4) and (5) [49].

(4) $$\log(q_{e,\mathrm{cal}}-q_{t})=-\frac{k}{2.303}t+\log q_{e,\mathrm{cal}}$$

(5) $$q=\frac{(C_{0}-Ce)V}{m}$$

where *q* (mg/g) is the adsorption amount for AO7 dye, *k* (1/h) is the apparent rate constant, *C*_0_ (mg/L) is the initial concentration at *t* = 0, *C*_e_ (mg/L) is the solution phase concentration of AO7, *m* (g) is the mass of CeO_2_ samples and *V* (L) is the volume of AO7 aqueous solution. The variations in log(*q*_e_-*q*_t_) as a function of illumination time are shown in Figure 9. The apparent rate constants (*k*) and relative coefficients (*R*^2^) obtained by fitting with the pseudo-first-order model are summarized in Table 2. It was found that the 2% Pr-doped CeO_2_ showed the highest degradation rate, and that was *k* of 2%; Pr-doped CeO_2_ was about 1.7 times higher than that of undoped CeO_2_. The enhancement of photocatalytic activity was attributed to the doping effect induced by Pr. In addition, the correlation coefficients (*R*^2^) of all samples are above 0.93, suggesting that the photocatalytic degradation process of the AO7 molecule tends to follow the pseudo-first-order kinetic model.

From the above results of pseudo-first-order kinetic analysis, the apparent rate constants (*k*) of the photocatalytic degradation of the dye in presence of 3% Pr-doped CeO_2_ was highest, suggesting that AO7 degrades faster under the irradiation of ultraviolet light. From Figure 8a, we could find that the removal rates of AO7 by 1% and 2% Pr-doped CeO_2_ approached 100% within 4 h of reaction. The degradation efficiency of 1% Pr-doped CeO_2_ for AO7 dye was evaluated by comparing it with those of other reported materials, as shown in Table 3. In consideration of the presented results in Table 3, it turned out that 1% Pr-doped CeO_2_ was also an alternative material for the degradation of AO7 dye.

**Figure 10:**
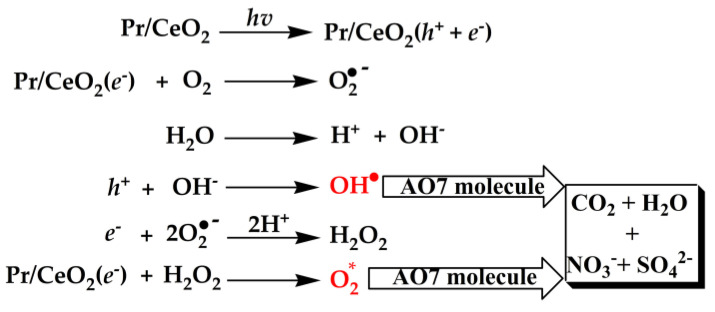
Proposed radical-ions mechanism for AO7 degradation.

Figure 10 shows the proposed radical ion mechanism for AO7 degradation. Under the irradiation of ultraviolet light, electron (*e*^-^) was excited from the VB to CB of Pr-doped CeO_2_ catalyst (Pr/CeO_2_) and a charge vacancy in the hole (*h*^+^) in the valence band was also created. These photogenerated *h*^+^ and *e*^-^ could react with the adsorbed water (H_2_O) and O_2_ on the surface of the Pr/CeO_2_ catalyst, the active oxidative ionic radicals (O_2_^•-^) and the hydroxyl radicals (OH^•^) generated in the reaction medium, which were very reactive and could quickly oxidize organic species on the Pr/CeO_2_ surface. Moreover, the generation of OH^•^ radicals along with the formation of hydrogen peroxides (H_2_O_2_) increased the catalytic activity towards the degradation of AO7. Subsequently, the generated H_2_O_2_ reacted with Pr/CeO_2_(*e*^-^), leading to the formation of nascent oxygen (active oxygen, O_2_*), which happened with the reduction of Ce^4+^ to Ce^3+^ states. The tetravalent oxidation state of Ce could provide multiple photogenerated *e*^-^ to vary the electrical conductivity and enhance the catalytic activity of Pr/CeO_2_. Furthermore, the Pr-doping could induce the formation of *V*_O_ defects in the CeO_2_ crystal, which could enhance its capacity to capture oxygen from the environment, and these stored oxygen species could be released quickly to the reaction medium, and then more highly reactive O_2_* species were produced, leading to the degradation of AO7 dye under the irradiation of ultraviolet light. Finaly, there was the degradation of the enlisted organics to CO_2_, H_2_O and other less toxic minerals such as nitrates and sulphates [58]. From the above description, the formation of the photogenerated *h*^+^ and *e*^-^ was the beginning of the catalytic reaction. If the CeO_2_ catalyst surface was completely covered by AO7 molecules, the generation of *h*^+^ and *e*^-^ would be seriously affected because of the blocked contact between ultraviolet light and the CeO_2_ catalyst surface, and the catalytic efficiency of the catalyst would also decrease.

## 4. Conclusions

Pr-doped CeO_2_ solid solutions were synthesized via a simple solvothermal process followed by calcination. The characterization results confirmed the successful doping of Pr elements with a trivalent state into a CeO_2_ lattice, and the solid solubility limit of Pr in the CeO_2_ lattice was recognized as 3%. The 6% Pr-doped CeO_2_ still retained a fluorite crystal structure and original multilayered mesoporous structure; however, Pr-doping could affect their specific surface areas. The Raman spectra revealed that Pr-doping was beneficial for the creation of oxygen vacancy defects, and the relative [*V*_O_]_Raman_ reached a maximum in 1% Pr-doped CeO_2_. Both 1% and 2% Pr-doped CeO_2_ were alternative photocatalysts for the degradation of AO7 dye, as they showed a maximum removal ability that approached 100% at room temperature and 0.1 g of catalyst within 4 h without pH preadjustment. Moreover, the photocatalytic degradation process of the AO7 molecule tends to follow the pseudo-first-order kinetic model. Hence, such Pr-doped mesoporous CeO_2_ has the potential for the treatment of dyestuff wastewater generated by the industry.

## Figures and Tables

**Table 1:** Practical Pr contents, grain size, lattice parameters and [*V*_O_]_XPS_ and [*V*_O_]_XPS_ of Undoped CeO_2_ and Pr-doped CeO_2_ with different Pr contents synthesized by solvothermal method at 200 °C for 24 h followed by calcination in air at 500 °C for 2 h.

Pr-Doped CeO_2_		Pr Contents (%)					
	Undoped	1.0	2.0	3.0	4.0	5.0	6.0
Practical Pr content (%)	/	0.92	2.11	3.06	3.92	5.23	5.59
Grain size (nm)	16.3	16.2	11.8	10.4	10.7	13.2	15.7
Lattice parameter (nm)	0.54157	0.54246	0.54213	0.54332	0.54278	0.54292	0.54235
[*V*_O_]_XPS_ (%)	24.36	33.30	31.67	/	/	/	/
[*V*_O_]_Raman_	0.676	0.813	0.785	0.777	0.761	0.751	0.752

**Table 2:** Kinetic parameters for the degradation of AO7 dye onto Undoped CeO_2_ and Pr-doped CeO_2_ under ultraviolet lamp.

Pr-Doped CeO_2_		Pr Contents (%)					
	Undoped	1.0	2.0	3.0	4.0	5.0	6.0
*k* (1/h)	0.0939	0.1239	0.1624	0.1292	0.0857	0.0896	0.0556
*R* ^2^	0.9775	0.9778	0.9599	0.9686	0.9631	0.9427	0.9332

**Table 3:** Recent literatures on the development for the degradation of AO7 dye.

Catalyst	[Catalyst]; [AO7]; *V*	Adsorption (%)	Light Source	Degradation (%)	Time (h)
TiO_2_ (P25) [50]	1.0 g/L; 40 ppm	~3	Two UV lamps (6 W; 365 nm)	~32	4
Mesoporous TiO_2_ nanotube [51]	1.0 g/L; 300 ppm; 200 mL (pH = 3)	~39	Immersed UVP Pen-Ray lamp (11 W; 254 nm)	100	3
TiO_2_ nanotube layers annealed at 500 °C [52]	− 5 × 10^−5^ mol/L; 15 mL	~32	Philips-TDL UV lamps (8 W; 350–400 nm)	~92	40
La^3+^-doped TiO_2_ [53]	4.0 g/L; 50 ppm; 500 mL	~18	Topbulb, F8T5/DL fluorescent daylight lamps (116 W)	79	6
4.0% WO_x_/TiO_2_ [54]	1.0 g/L; 25 ppm; 100 mL	~35	Halogen lamp with 20,000 lm luminescence (1000 W; 420 nm)	100	4
ZnCr-SO_4_ [48]	0.4g/L; 50 ppm; 50 mL	~25	Ultra-Vitalux lamp (300 W)	~66	2
ZnCr-CO_3_ [55]	0.5 g/L; 5 × 10^−5^ mol/L; 60 mL	~6	Philips HPW high-pressure mercury lamp (125 W; 365 nm)	~66	5
BiOBr/32% PBCD-B-D [56]	1.0 g/L; 0.2 mM; 40 mL	~55	Xenon lamp (500 W; 420 nm)	92.1	6
CeO_2_ nanopaticles [57]	1.0 g/L; 50 ppm; 50 mL (pH = 6.8)	~25	Halogen-tungsten lamp (1000 W; <420 nm)	98	11
1% Pr-doped CeO_2_ in this work	1.0 g/L; 20 ppm; 100mL	40.2	Medical ultraviolet disinfection lamp (300 W; 254 nm)	~100	4

## Data Availability

Not applicable.
